# (Carbonato-κ^2^ *O*,*O*′)bis­(di-2-pyridyl­amine-κ^2^ *N*,*N*′)cobalt(III) bromide

**DOI:** 10.1107/S1600536811008051

**Published:** 2011-03-09

**Authors:** Agnieszka Czapik, Christos Papadopoulos, Maria Lalia-Kantouri, Maria Gdaniec

**Affiliations:** a Faculty of Chemistry, Adam Mickiewicz University, 60-780 Poznań, Poland; b Department of Chemistry, Aristotle University of Thessaloniki, Thessaloniki 54124, Greece

## Abstract

In the title compound, [Co(CO_3_)(C_10_H_9_N_3_)_2_]Br, a distorted octa­hedral coordination of the Co^III^ atom is completed by four N atoms of the two chelating di-2-pyridyl­amine ligands and two O atoms of the chelating carbonate anion. The di-2-pyridyl­amine ligands are nonplanar and the dihedral angles between the 2-pyridyl groups are 29.11 (9) and 37.15 (12)°. The coordination cation, which has approximate *C*_2_ symmetry, is connected to the bromide ion *via* an N—H⋯Br^−^ hydrogen bond. The ionic pair thus formed is further assembled into a dimer *via* N—H⋯O inter­actions about an inversion centre. A set of weaker C—H⋯O and C—H⋯Br^−^ inter­actions connect the dimers into a three-dimensional network.

## Related literature

For the crystal structure of the isostructural [bis­(di-2-pyridyl­amine-κ^2^ *N*,*N*′)](carbonato-κ^2^ *O*,*O*′)cobalt(III) nitrate, see: Castillo *et al.* (2011 ▶). For the crystal structure of the perchlorate salt, see: Williams *et al.* (1987 ▶).
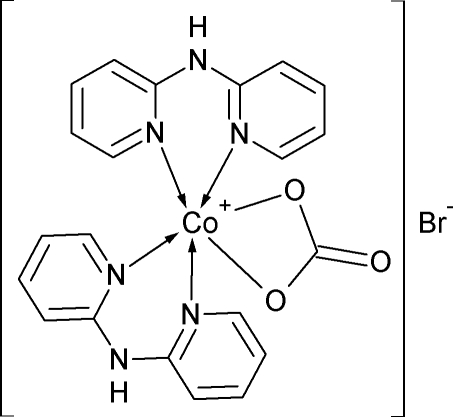


## Experimental

### 

#### Crystal data

[Co(CO_3_)(C_10_H_9_N_3_)_2_]Br
*M* *_r_* = 541.25
Monoclinic, 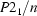

*a* = 16.9605 (3) Å
*b* = 7.4322 (1) Å
*c* = 17.2590 (4) Å
β = 105.839 (2)°
*V* = 2092.96 (7) Å^3^
*Z* = 4
Mo *K*α radiation
μ = 2.77 mm^−1^
*T* = 130 K
0.30 × 0.15 × 0.05 mm

#### Data collection

Oxford Diffraction Xcalibur E diffractometer
Absorption correction: multi-scan (*CrysAlis PRO*; Agilent Technologies, 2010 ▶) *T*_min_ = 0.710, *T*_max_ = 0.874
29846 measured reflections
4277 independent reflections
3386 reflections with *I* > 2σ(*I*)
*R*_int_ = 0.064

#### Refinement

*R*[*F* ^2^ > 2σ(*F* ^2^)] = 0.037
*wR*(*F* ^2^) = 0.080
*S* = 1.01
4277 reflections
289 parameters
H-atom parameters constrained
Δρ_max_ = 0.79 e Å^−3^
Δρ_min_ = −0.50 e Å^−3^

### 

Data collection: *CrysAlis PRO* (Agilent Technologies, 2010 ▶); cell refinement: *CrysAlis PRO*; data reduction: *CrysAlis PRO*; program(s) used to solve structure: *SHELXS97* (Sheldrick, 2008 ▶); program(s) used to refine structure: *SHELXL97* (Sheldrick, 2008 ▶); molecular graphics: *ORTEP-3 for Windows* (Farrugia, 1997 ▶) and *Mercury* (Macrae *et al.*, 2006 ▶); software used to prepare material for publication: *SHELXL97*.

## Supplementary Material

Crystal structure: contains datablocks global, I. DOI: 10.1107/S1600536811008051/su2260sup1.cif

Structure factors: contains datablocks I. DOI: 10.1107/S1600536811008051/su2260Isup2.hkl

Additional supplementary materials:  crystallographic information; 3D view; checkCIF report

## Figures and Tables

**Table 1:** Selected bond lengths (Å)

Co1—O2	1.901 (2)
Co1—O1	1.904 (2)
Co1—N9*B*	1.919 (2)
Co1—N1*A*	1.923 (2)
Co1—N1*B*	1.925 (2)
Co1—N9*A*	1.933 (2)

**Table 2:** Hydrogen-bond geometry (Å, °)

*D*—H⋯*A*	*D*—H	H⋯*A*	*D*⋯*A*	*D*—H⋯*A*
N7*A*—H7*A*⋯O2^i^	0.86	2.08	2.876 (3)	154
N7*B*—H7*B*⋯Br1	0.86	2.49	3.327 (2)	164
C10*B*—H10*B*⋯O3^ii^	0.95	2.34	3.225 (4)	155
C5*A*—H5*A*⋯O1^iii^	0.95	2.44	3.265 (4)	146
C13*A*—H13*A*⋯O3^i^	0.95	2.43	3.316 (4)	156
C13*B*—H13*B*⋯Br1	0.95	2.83	3.623 (3)	142
C5*B*—H5*B*⋯Br1^iv^	0.95	2.86	3.741 (3)	155
C4*B*—H4*B*⋯Br1^v^	0.95	2.89	3.657 (3)	139

Symmetry codes: (i) 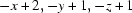; (ii) 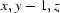; (iii) 
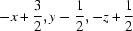
; (iv) 
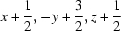
; (v) 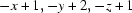.

**Fig. 1.**
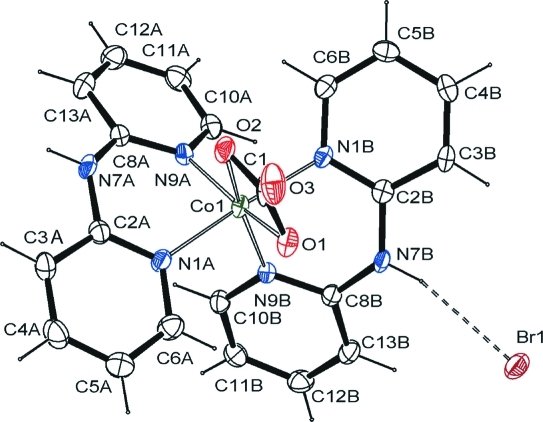
Asymmetric unit of the title compound with the displacement ellipsoids shown at the 50% probability level.

**Fig. 2.**
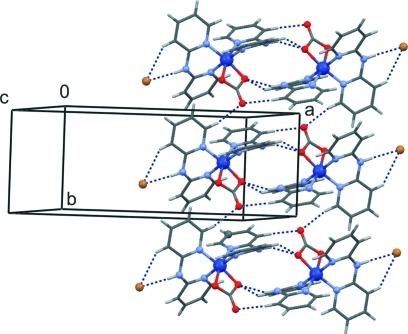
Centrosymmetric dimers of the ionic pairs of the title compound connected via C10B—H10B···O3 interaction into chains extended along [0 1 0]. N, O, Br and Co atoms are shown as spheres of arbitrary radii. Short contacts and hydrogen bonds are shown with dashed lines.

## supplementary crystallographic information

### Comment

The title compound (Fig. 1) was obtained as a byproduct in the preparation of [Co(Hdpa)_2_(apo)]Br from CoBr_2_, where apo is 2-acetylphenolate ion and Hdpa is di-2-pyridylamine. The chelating carbonate ligand in the coordination cation was identified from its geometrical parameters, namely the two bonds of 1.325 (4) and 1.341 (4) Å indicated single C—O bonds and the bond length of 1.183 (4) Å pointed to a C=O bond. The presence of the carbonate anion in the studied compound concurred with the +3 oxidation state of the cobalt atom. The Co^III^ atom shows a distorted octahedral coordination that is completed by four N atoms of the two chelating di-2-pyridylamine ligands and two O atoms of the chelating carbonate anion (Table 1). The bidentate Hdpa ligands chelate the Co^III^ atom to form two six-membered rings with the N1—Co1—N9 bite angles of 90.42 (10) and 88.48 (10)°, in ligand A and B respectively. The diimine ligands are non-planar with the N(py)—C—N(H)—C torsion angles of 28.1 (4) and -27.6 (4)° in the A ligand and 28.0 (4) and -33.5 (4)° in the B Hdpa ligand.

The coordination cation has approximate C_2_ symmetry with a pseudo-twofold axis passing through atoms O3, C1 and Co1. It binds one Br^-^ anion *via* a N—H···Br^-^ hydrogen bond and the ionic pair thus formed assembles into a dimer, *via* N—H···O hydrogen bonds (Fig. 2, Table 2), centered about an inversion center. These dimers are further connected *via* a C10B—H10B···O3(*x*, *y* - 1, *z*) interaction into chains extended along [010] and the chains are joined *via* a set of C—H···O and C—H···Br^-^ interactions into a three-dimensional network (Table 2).

Interesingly, similar interactions between the coordination cation and the anion, and the formation of dimers of the ionic pairs, was also observed in the nitrate and perchlorate salts of the same cation (Castillo *et al.*, 2011; Williams *et al.*, 1987). Moreover, the crystals of the nitrate salt and the title bromide salt are to a large extent isostructural.

### Experimental

2 mmol (0.342 g) of di-2-pyridylamine (Hdpam), dissolved in a small amount of EtOH, was added to a solution of 1 mmol (0.238 g) of CoBr_2_.H_2_O in 5 ml of EtOH and the mixture was stirred for 30 min. Then, to this mixture was added dropwise an ethanolic solution containing 1 mmol (0.136 g) of 2-hydroxyacetophenone (Hapo) and 1 mmol of CH_3_ONa. The solution was stirred at room temperature under an argon atmosphere for 2 h or refluxed with continuous stirring. Two types of crystals precipitated from the solution. The main product, in the form of small crystals of light orange color, was identified as the mixed-ligand Co^II^ complex [Co(dpamH)_2_(apo)]Br (mean yield 62%). The side-product, in the form of large dark-red crystals, was identified as the Co^III^complex, [Co(dpamH)_2_(CO_3_)]Br. When the reaction between CoBr_2_ and Hdpam was repeated in air the title compound, [Co(dpamH)_2_(CO_3_)]Br, was obtained as the main product.

### Refinement

The H-atoms of the NH groups were located in difference electron-density maps. In the final cycles of lease-squares refinement the N—H bond lengths were constrained to 0.86 Å with *U*_iso_(H) = 1.2*U*_eq_(N). All the other H-atoms were initially identified in difference electron-density maps but were placed at calculated positions, with C—H = 0.95 Å, and were refined as riding on their carrier atoms, with *U*_iso_(H) = 1.2*U*_eq_(C).

### Crystal data

**Table:**

[Co(CO_3_)(C_10_H_9_N_3_)_2_]Br	*F*(000) = 1088
*M**_r_* = 541.25	*D*_x_ = 1.718 Mg m^−^^3^
Monoclinic, *P*2_1_/*n*	Mo *K*α radiation, λ = 0.71073 Å
Hall symbol: -P 2yn	Cell parameters from 8233 reflections
*a* = 16.9605 (3) Å	θ = 3.0–30.3°
*b* = 7.4322 (1) Å	µ = 2.77 mm^−^^1^
*c* = 17.2590 (4) Å	*T* = 130 K
β = 105.839 (2)°	Plate, red
*V* = 2092.96 (7) Å^3^	0.30 × 0.15 × 0.05 mm
*Z* = 4	

### Data collection

**Table:**

Oxford Diffraction Xcalibur E diffractometer	4277 independent reflections
Radiation source: fine-focus sealed tube	3386 reflections with *I* > 2σ(*I*)
graphite	*R*_int_ = 0.064
Detector resolution: 16.1544 pixels mm^-1^	θ_max_ = 26.4°, θ_min_ = 3.0°
ω scans	*h* = −21→21
Absorption correction: multi-scan (*CrysAlis PRO*; Agilent Technologies, 2010)	*k* = −9→9
*T*_min_ = 0.710, *T*_max_ = 0.874	*l* = −21→21
29846 measured reflections	

### Refinement

**Table:**

Refinement on *F*^2^	Primary atom site location: structure-invariant direct methods
Least-squares matrix: full	Secondary atom site location: difference Fourier map
*R*[*F*^2^ > 2σ(*F*^2^)] = 0.037	Hydrogen site location: inferred from neighbouring sites
*wR*(*F*^2^) = 0.080	H-atom parameters constrained
*S* = 1.01	*w* = 1/[σ^2^(*F*_o_^2^) + (0.0316*P*)^2^] where *P* = (*F*_o_^2^ + 2*F*_c_^2^)/3
4277 reflections	(Δ/σ)_max_ = 0.001
289 parameters	Δρ_max_ = 0.79 e Å^−^^3^
0 restraints	Δρ_min_ = −0.50 e Å^−^^3^

### Special details

**Table:**

Geometry. All e.s.d.'s (except the e.s.d. in the dihedral angle between two l.s. planes) are estimated using the full covariance matrix. The cell e.s.d.'s are taken into account individually in the estimation of e.s.d.'s in distances, angles and torsion angles; correlations between e.s.d.'s in cell parameters are only used when they are defined by crystal symmetry. An approximate (isotropic) treatment of cell e.s.d.'s is used for estimating e.s.d.'s involving l.s. planes.
Refinement. Refinement of *F*^2^ against ALL reflections. The weighted *R*-factor *wR* and goodness of fit *S* are based on *F*^2^, conventional *R*-factors *R* are based on *F*, with *F* set to zero for negative *F*^2^. The threshold expression of *F*^2^ > σ(*F*^2^) is used only for calculating *R*-factors(gt) *etc*. and is not relevant to the choice of reflections for refinement. *R*-factors based on *F*^2^ are statistically about twice as large as those based on *F*, and *R*- factors based on ALL data will be even larger.

### Fractional atomic coordinates and isotropic or equivalent isotropic displacement parameters (Å^2^)

**Table:**

	*x*	*y*	*z*	*U*_iso_*/*U*_eq_	
Co1	0.79115 (2)	0.47425 (5)	0.48500 (2)	0.01679 (12)	
Br1	0.42522 (2)	0.71322 (4)	0.36946 (2)	0.02940 (11)	
N1A	0.83606 (15)	0.3707 (3)	0.40419 (14)	0.0189 (6)	
C2A	0.91367 (19)	0.3175 (4)	0.42120 (18)	0.0203 (7)	
C3A	0.9525 (2)	0.2829 (4)	0.36081 (19)	0.0255 (8)	
H3A	1.0091	0.2535	0.3741	0.031*	
C4A	0.9066 (2)	0.2925 (4)	0.2818 (2)	0.0305 (8)	
H4A	0.9311	0.2677	0.2396	0.037*	
C5A	0.8242 (2)	0.3387 (4)	0.2639 (2)	0.0303 (8)	
H5A	0.7914	0.3429	0.2097	0.036*	
C6A	0.7916 (2)	0.3778 (4)	0.32558 (18)	0.0261 (8)	
H6A	0.7355	0.4114	0.3134	0.031*	
N7A	0.95794 (15)	0.2983 (3)	0.50046 (15)	0.0223 (6)	
H7A	1.0091	0.2787	0.5059	0.027*	
C8A	0.92811 (19)	0.2571 (4)	0.56488 (18)	0.0193 (7)	
N9A	0.85087 (15)	0.3025 (3)	0.56334 (14)	0.0171 (6)	
C10A	0.8212 (2)	0.2466 (4)	0.62506 (18)	0.0211 (7)	
H10A	0.7656	0.2708	0.6227	0.025*	
C11A	0.8687 (2)	0.1567 (4)	0.69034 (19)	0.0253 (8)	
H11A	0.8469	0.1205	0.7331	0.030*	
C12A	0.9494 (2)	0.1196 (4)	0.6927 (2)	0.0287 (8)	
H12A	0.9838	0.0598	0.7381	0.034*	
C13A	0.9796 (2)	0.1685 (4)	0.63018 (19)	0.0266 (8)	
H13A	1.0347	0.1426	0.6313	0.032*	
N1B	0.74231 (14)	0.5957 (3)	0.55895 (14)	0.0169 (5)	
C2B	0.66482 (18)	0.6546 (4)	0.53146 (17)	0.0171 (7)	
C3B	0.63533 (19)	0.7949 (4)	0.56985 (18)	0.0195 (7)	
H3B	0.5827	0.8453	0.5464	0.023*	
C4B	0.68320 (19)	0.8582 (4)	0.64138 (19)	0.0223 (7)	
H4B	0.6641	0.9530	0.6685	0.027*	
C5B	0.76043 (19)	0.7830 (4)	0.67458 (19)	0.0219 (7)	
H5B	0.7926	0.8179	0.7265	0.026*	
C6B	0.78852 (19)	0.6586 (4)	0.63082 (18)	0.0202 (7)	
H6B	0.8427	0.6139	0.6514	0.024*	
N7B	0.61387 (15)	0.5775 (3)	0.46391 (14)	0.0182 (6)	
H7B	0.5646	0.6183	0.4492	0.022*	
C8B	0.61995 (18)	0.4030 (4)	0.43871 (17)	0.0173 (7)	
N9B	0.69443 (15)	0.3281 (3)	0.45216 (14)	0.0170 (6)	
C10B	0.69889 (19)	0.1502 (4)	0.43516 (18)	0.0203 (7)	
H10B	0.7512	0.0943	0.4474	0.024*	
C11B	0.63142 (19)	0.0493 (4)	0.40141 (18)	0.0219 (7)	
H11B	0.6365	−0.0754	0.3917	0.026*	
C12B	0.5554 (2)	0.1309 (4)	0.38152 (18)	0.0248 (7)	
H12B	0.5078	0.0643	0.3553	0.030*	
C13B	0.54920 (19)	0.3088 (4)	0.39994 (18)	0.0224 (7)	
H13B	0.4974	0.3670	0.3865	0.027*	
C1	0.82969 (19)	0.7580 (4)	0.4471 (2)	0.0224 (7)	
O1	0.75759 (13)	0.6760 (3)	0.41586 (12)	0.0240 (5)	
O2	0.87502 (12)	0.6516 (3)	0.50343 (12)	0.0229 (5)	
O3	0.85043 (14)	0.8971 (3)	0.42570 (15)	0.0363 (6)	

### Atomic displacement parameters (Å^2^)

**Table:**

	*U*^11^	*U*^22^	*U*^33^	*U*^12^	*U*^13^	*U*^23^
Co1	0.0119 (2)	0.0186 (2)	0.0203 (2)	0.00264 (17)	0.00510 (18)	0.00095 (17)
Br1	0.02154 (19)	0.02402 (18)	0.0356 (2)	0.00546 (14)	−0.00408 (15)	−0.00196 (15)
N1A	0.0141 (14)	0.0245 (14)	0.0179 (14)	0.0031 (11)	0.0042 (11)	0.0019 (11)
C2A	0.0197 (18)	0.0205 (16)	0.0220 (17)	0.0004 (13)	0.0077 (14)	−0.0015 (13)
C3A	0.0230 (19)	0.0280 (18)	0.0289 (19)	0.0047 (14)	0.0126 (16)	−0.0012 (15)
C4A	0.035 (2)	0.036 (2)	0.0261 (19)	0.0044 (16)	0.0169 (17)	−0.0010 (15)
C5A	0.032 (2)	0.036 (2)	0.0225 (19)	0.0023 (16)	0.0063 (16)	−0.0009 (15)
C6A	0.0230 (19)	0.0319 (19)	0.0231 (18)	0.0001 (15)	0.0058 (15)	0.0038 (15)
N7A	0.0114 (14)	0.0331 (15)	0.0230 (15)	0.0053 (11)	0.0054 (12)	−0.0009 (12)
C8A	0.0167 (17)	0.0209 (16)	0.0203 (17)	0.0022 (13)	0.0051 (14)	−0.0042 (13)
N9A	0.0154 (14)	0.0182 (13)	0.0187 (14)	0.0019 (10)	0.0063 (11)	−0.0018 (10)
C10A	0.0220 (18)	0.0178 (16)	0.0250 (17)	0.0011 (13)	0.0089 (15)	−0.0016 (13)
C11A	0.035 (2)	0.0195 (16)	0.0222 (18)	0.0013 (15)	0.0089 (16)	0.0003 (14)
C12A	0.031 (2)	0.0256 (18)	0.0242 (18)	0.0072 (15)	−0.0007 (16)	0.0011 (15)
C13A	0.0202 (18)	0.0299 (18)	0.0278 (19)	0.0079 (15)	0.0030 (15)	0.0021 (15)
N1B	0.0115 (13)	0.0173 (13)	0.0213 (14)	0.0012 (10)	0.0036 (11)	0.0011 (11)
C2B	0.0174 (17)	0.0142 (14)	0.0207 (16)	−0.0019 (12)	0.0070 (14)	0.0035 (13)
C3B	0.0153 (17)	0.0192 (16)	0.0260 (18)	0.0053 (13)	0.0091 (14)	0.0041 (13)
C4B	0.0259 (19)	0.0166 (15)	0.0278 (18)	0.0020 (14)	0.0132 (15)	−0.0017 (14)
C5B	0.0258 (19)	0.0194 (16)	0.0195 (17)	−0.0048 (14)	0.0044 (15)	−0.0025 (13)
C6B	0.0163 (17)	0.0189 (15)	0.0240 (17)	−0.0002 (13)	0.0035 (14)	0.0019 (13)
N7B	0.0106 (13)	0.0199 (13)	0.0233 (14)	0.0047 (10)	0.0032 (11)	−0.0007 (11)
C8B	0.0155 (16)	0.0186 (15)	0.0179 (16)	0.0014 (13)	0.0047 (13)	0.0010 (13)
N9B	0.0139 (14)	0.0198 (13)	0.0173 (13)	0.0023 (11)	0.0045 (11)	−0.0018 (11)
C10B	0.0202 (18)	0.0196 (16)	0.0210 (17)	0.0049 (13)	0.0057 (14)	0.0005 (13)
C11B	0.0259 (19)	0.0180 (16)	0.0230 (17)	−0.0009 (14)	0.0086 (15)	−0.0017 (13)
C12B	0.0213 (18)	0.0264 (18)	0.0253 (18)	−0.0059 (14)	0.0040 (15)	−0.0047 (14)
C13B	0.0139 (17)	0.0256 (18)	0.0268 (18)	0.0003 (13)	0.0039 (14)	−0.0014 (14)
C1	0.0176 (18)	0.0240 (18)	0.0320 (19)	0.0082 (14)	0.0175 (15)	0.0041 (15)
O1	0.0208 (13)	0.0246 (12)	0.0286 (13)	0.0063 (10)	0.0099 (10)	0.0058 (10)
O2	0.0129 (11)	0.0253 (11)	0.0314 (13)	0.0001 (9)	0.0074 (10)	−0.0032 (10)
O3	0.0330 (15)	0.0249 (13)	0.0612 (17)	0.0006 (11)	0.0300 (13)	0.0030 (12)

### Geometric parameters (Å, °)

**Table:**

Co1—O2	1.901 (2)	C13A—H13A	0.9500
Co1—O1	1.904 (2)	N1B—C2B	1.343 (4)
Co1—N9B	1.919 (2)	N1B—C6B	1.357 (4)
Co1—N1A	1.923 (2)	C2B—N7B	1.373 (4)
Co1—N1B	1.925 (2)	C2B—C3B	1.399 (4)
Co1—N9A	1.933 (2)	C3B—C4B	1.363 (4)
N1A—C2A	1.328 (4)	C3B—H3B	0.9500
N1A—C6A	1.362 (4)	C4B—C5B	1.395 (4)
C2A—N7A	1.376 (4)	C4B—H4B	0.9500
C2A—C3A	1.401 (4)	C5B—C6B	1.360 (4)
C3A—C4A	1.375 (4)	C5B—H5B	0.9500
C3A—H3A	0.9500	C6B—H6B	0.9500
C4A—C5A	1.389 (5)	N7B—C8B	1.380 (4)
C4A—H4A	0.9500	N7B—H7B	0.8599
C5A—C6A	1.358 (4)	C8B—N9B	1.341 (4)
C5A—H5A	0.9500	C8B—C13B	1.393 (4)
C6A—H6A	0.9500	N9B—C10B	1.361 (4)
N7A—C8A	1.376 (4)	C10B—C11B	1.359 (4)
N7A—H7A	0.8600	C10B—H10B	0.9500
C8A—N9A	1.346 (4)	C11B—C12B	1.380 (4)
C8A—C13A	1.389 (4)	C11B—H11B	0.9500
N9A—C10A	1.362 (4)	C12B—C13B	1.371 (4)
C10A—C11A	1.367 (4)	C12B—H12B	0.9500
C10A—H10A	0.9500	C13B—H13B	0.9500
C11A—C12A	1.386 (5)	C1—O3	1.183 (4)
C11A—H11A	0.9500	C1—O2	1.325 (4)
C12A—C13A	1.364 (4)	C1—O1	1.341 (4)
C12A—H12A	0.9500		
			
O2—Co1—O1	68.96 (9)	C12A—C13A—C8A	118.7 (3)
O2—Co1—N9B	169.04 (10)	C12A—C13A—H13A	120.7
O1—Co1—N9B	100.10 (10)	C8A—C13A—H13A	120.7
O2—Co1—N1A	88.42 (10)	C2B—N1B—C6B	118.4 (3)
O1—Co1—N1A	88.32 (10)	C2B—N1B—Co1	118.4 (2)
N9B—Co1—N1A	92.01 (10)	C6B—N1B—Co1	121.5 (2)
O2—Co1—N1B	90.10 (9)	N1B—C2B—N7B	119.5 (3)
O1—Co1—N1B	86.40 (9)	N1B—C2B—C3B	121.2 (3)
N9B—Co1—N1B	88.48 (10)	N7B—C2B—C3B	119.3 (3)
N1A—Co1—N1B	174.69 (10)	C4B—C3B—C2B	119.0 (3)
O2—Co1—N9A	96.62 (10)	C4B—C3B—H3B	120.5
O1—Co1—N9A	165.55 (10)	C2B—C3B—H3B	120.5
N9B—Co1—N9A	94.32 (10)	C3B—C4B—C5B	119.6 (3)
N1A—Co1—N9A	90.42 (10)	C3B—C4B—H4B	120.2
N1B—Co1—N9A	94.81 (10)	C5B—C4B—H4B	120.2
C2A—N1A—C6A	118.5 (3)	C6B—C5B—C4B	118.5 (3)
C2A—N1A—Co1	121.6 (2)	C6B—C5B—H5B	120.8
C6A—N1A—Co1	119.1 (2)	C4B—C5B—H5B	120.8
N1A—C2A—N7A	119.3 (3)	N1B—C6B—C5B	122.6 (3)
N1A—C2A—C3A	122.0 (3)	N1B—C6B—H6B	118.7
N7A—C2A—C3A	118.7 (3)	C5B—C6B—H6B	118.7
C4A—C3A—C2A	118.3 (3)	C2B—N7B—C8B	125.2 (2)
C4A—C3A—H3A	120.9	C2B—N7B—H7B	116.0
C2A—C3A—H3A	120.9	C8B—N7B—H7B	112.9
C3A—C4A—C5A	119.8 (3)	N9B—C8B—N7B	118.9 (3)
C3A—C4A—H4A	120.1	N9B—C8B—C13B	121.5 (3)
C5A—C4A—H4A	120.1	N7B—C8B—C13B	119.7 (3)
C6A—C5A—C4A	118.6 (3)	C8B—N9B—C10B	118.1 (2)
C6A—C5A—H5A	120.7	C8B—N9B—Co1	120.32 (19)
C4A—C5A—H5A	120.7	C10B—N9B—Co1	121.3 (2)
C5A—C6A—N1A	122.6 (3)	N9B—C10B—C11B	122.6 (3)
C5A—C6A—H6A	118.7	N9B—C10B—H10B	118.7
N1A—C6A—H6A	118.7	C11B—C10B—H10B	118.7
C2A—N7A—C8A	127.2 (3)	C10B—C11B—C12B	118.9 (3)
C2A—N7A—H7A	112.7	C10B—C11B—H11B	120.5
C8A—N7A—H7A	117.3	C12B—C11B—H11B	120.5
N9A—C8A—N7A	119.9 (3)	C13B—C12B—C11B	119.5 (3)
N9A—C8A—C13A	121.9 (3)	C13B—C12B—H12B	120.3
N7A—C8A—C13A	118.2 (3)	C11B—C12B—H12B	120.3
C8A—N9A—C10A	118.3 (3)	C12B—C13B—C8B	119.1 (3)
C8A—N9A—Co1	120.1 (2)	C12B—C13B—H13B	120.5
C10A—N9A—Co1	120.9 (2)	C8B—C13B—H13B	120.5
N9A—C10A—C11A	122.2 (3)	O3—C1—O2	126.0 (3)
N9A—C10A—H10A	118.9	O3—C1—O1	126.0 (3)
C11A—C10A—H10A	118.9	O2—C1—O1	107.9 (3)
C10A—C11A—C12A	118.5 (3)	O3—C1—Co1	177.2 (3)
C10A—C11A—H11A	120.8	O2—C1—Co1	53.86 (14)
C12A—C11A—H11A	120.8	O1—C1—Co1	54.00 (14)
C13A—C12A—C11A	120.3 (3)	C1—O1—Co1	91.28 (17)
C13A—C12A—H12A	119.8	C1—O2—Co1	91.90 (18)
C11A—C12A—H12A	119.8		
			
N1A—C2A—N7A—C8A	28.1 (4)	N1B—C2B—N7B—C8B	28.0 (4)
N9A—C8A—N7A—C2A	−27.6 (4)	N9B—C8B—N7B—C2B	−33.5 (4)

### Hydrogen-bond geometry (Å, °)

**Table:**

*D*—H···*A*	*D*—H	H···*A*	*D*···*A*	*D*—H···*A*
N7A—H7A···O2^i^	0.86	2.08	2.876 (3)	154
N7B—H7B···Br1	0.86	2.49	3.327 (2)	164
C10B—H10B···O3^ii^	0.95	2.34	3.225 (4)	155
C5A—H5A···O1^iii^	0.95	2.44	3.265 (4)	146
C13A—H13A···O3^i^	0.95	2.43	3.316 (4)	156
C13B—H13B···Br1	0.95	2.83	3.623 (3)	142
C5B—H5B···Br1^iv^	0.95	2.86	3.741 (3)	155
C4B—H4B···Br1^v^	0.95	2.89	3.657 (3)	139

Symmetry codes: (i) −*x*+2, −*y*+1, −*z*+1; (ii) *x*, *y*−1, *z*; (iii) −*x*+3/2, *y*−1/2, −*z*+1/2; (iv) *x*+1/2, −*y*+3/2, *z*+1/2; (v) −*x*+1, −*y*+2, −*z*+1.

## References

[bb1] Agilent Technologies (2010). *CrysAlis PRO.* Agilent Technologies, Yarnton, Oxfordshire, England.

[bb2] Castillo, O., Luque, A., De la Pinta, N. & Román, P. (2011). *Acta Cryst.* E**67**, e15. 10.1107/S1600536810050798 PMC3052119 21522228

[bb3] Farrugia, L. J. (1997). *J. Appl. Cryst.* **30**, 565.

[bb4] Macrae, C. F., Edgington, P. R., McCabe, P., Pidcock, E., Shields, G. P., Taylor, R., Towler, M. & van de Streek, J. (2006). *J. Appl. Cryst.* **39**, 453–457.

[bb5] Sheldrick, G. M. (2008). *Acta Cryst.* A**64**, 112–122. 10.1107/S0108767307043930 18156677

[bb6] Williams, A. F., Bocquet, B. & Bernardinelli, G. (1987). *Acta Cryst.* C**43**, 883–885.

